# Management of outpatient parenteral antibiotic therapy: a United States-based multi-center survey

**DOI:** 10.1017/ash.2026.10347

**Published:** 2026-04-17

**Authors:** Laila M. Castellino, Christina G. Rivera, Susan E. Beekmann, Philip M. Polgreen, Monica M. Mahoney, Sara C. Keller

**Affiliations:** 1 The University of Texas Southwestern Medical Center and Parkland Health, Dallas, TX, USA; 2 Mayo Clinic, Rochester, MN, USA; 3 The University of Iowa, Iowa City, IA, USA; 4 Beth Israel Deaconess Medical Center, Boston, MA, USA; 5 Johns Hopkins University School of Medicine, Baltimore, MD, USA

## Abstract

**Objective:**

Outpatient parenteral antimicrobial therapy (OPAT) programs continue to evolve with increases in multidisciplinary teams, complex oral antibiotics, long-acting injectable antimicrobials, patients with substance use disorder (SUD) and telehealth. We sought to examine how OPAT programs are currently structured and identify barriers to safe care.

**Design:**

Cross-sectional survey.

**Participants:**

Physician, pharmacist, and advanced practice providers (APP) Emerging Infections Network (EIN) members.

**Methods:**

A survey was conducted between February and March 2025 to assess OPAT structure, expansion to complex outpatient antimicrobial therapy (COpAT), and barriers to safe OPAT care.

**Results:**

Of 1639 EIN members, 622 (38%) responded; 75% reported an active role in OPAT. Oversight of patients on COpAT was reported by 59%, and mandatory ID consultation for OPAT by 59%. Eighty-one percent reported >75% of OPAT patients were followed by ID. Most patients received OPAT at home (78%) followed by post-acute care facilities. Outpatient and inpatient ID physicians were responsible for laboratory test monitoring (75% and 30% respectively), while 37% reported a multidisciplinary OPAT team and 32% reported non-specialists. Respondents cited inadequate support in data analysis, administration, information technology and financial support for clinical staff. Common challenges were lack of leadership awareness (51%), difficulty managing patients with SUD (50%) and timely access to laboratory test results (48%).

**Conclusions:**

ID physicians were commonly involved in OPAT care, with many programs expanding to COpAT. Several barriers to the safe delivery of OPAT remain, including lack of institutional support, reimbursement and poor communication between stakeholders.

## Introduction

Outpatient parenteral antimicrobial therapy (OPAT) is a safe and cost-effective way for patients to continue to receive intravenous (IV) antimicrobials outside the hospital setting.^1^ Patients appreciate the improved quality of life while continuing to receive care in their home environment.^2^ Guidelines outline best practices for patients receiving OPAT,^1,3^ and dedicated OPAT teams have been shown to decrease overall healthcare costs by decreasing hospital length of stay, readmission rates and emergency department visits.^4,5^ With increased recognition of the value of OPAT, hospitals, health systems and physician practices have developed multidisciplinary OPAT teams to ensure the safe transition of care from inpatient to ambulatory care.^6^

Despite the many benefits and value of dedicated OPAT teams, no standards exist for what constitutes an OPAT team, and how best to support a program. Additionally, in keeping with new evidence, many Infectious Diseases (ID) clinicians are increasingly using high-risk oral antimicrobials and long-acting injectable antimicrobials in place of traditional IV antimicrobials and taking innovative approaches to the care of patients with substance use disorder (SUD) to best support successful patient outcomes.^7–10^ Several publications have demonstrated approaches incorporating electronic health records (EHR) to streamline patient management and follow-up,^11,12^ as well as capture workload associated with OPAT management.^13^ Reimbursement structures have also changed, as have methods of following OPAT patients, with increasing use of telehealth, advanced practice providers (APPs) and ID pharmacists.^14,15^ Given this changing healthcare landscape, we sought to examine how OPAT programs have evolved to encompass changes in healthcare delivery, as well as understand the ongoing barriers to optimal OPAT care among US physicians, pharmacists, and APPs.

The Infectious Diseases Society of America (IDSA) Emerging Infections Network (EIN) is a provider-based sentinel network supported by the Centers for Disease Control and Prevention (CDC).^16^ Membership includes ID physicians, ID pharmacists, ID APPs, and public health professionals. Whereas previous EIN surveys focused on physicians, the current survey also included pharmacists and APPs, to understand how the practice of OPAT has evolved since the last survey in 2018.^17–19^

## Methods

### Instrument

We developed a survey instrument in collaboration with ID physicians, ID pharmacists, and EIN staff. The instrument contained 12 multiple-choice or Likert items and one free-text item allowing respondents to provide additional comments. The survey was piloted with a group of content experts, and questions were modified based on their feedback. The survey focused on the respondents’ role with OPAT, structure of OPAT provision, location where OPAT is received, providers responsible for managing OPAT, time devoted to OPAT, institutional support given to OPAT, inclusion of high-risk oral-only and long-acting injectable antimicrobials [complex outpatient antimicrobial therapy (COpAT)] within OPAT structures, oversight of OPAT, and barriers to safe OPAT care (Appendix 1). Respondents did not have to answer every question, and hence the results are reported based on the denominator of respondents who answered a particular question. The University of Iowa Institutional Review Board (IRB) determined that the project was deemed non-human subjects research and was thus IRB exempt.

### Study population and distribution

The IDSA EIN listserv (electronic mailing list) uses email to disseminate daily (Monday through Friday) ID, clinical, and epidemiologic observations and replies for its membership. The EIN was established as a provider-based emerging infections surveillance network that communicates regularly about emerging infectious diseases and related phenomena. EIN members who are physicians, pharmacists or APPs also volunteer to complete surveys. Baseline geographic and practice characteristics for EIN members are maintained in a database and updated regularly.

Members of the EIN were sent a link to a confidential survey between February 25 and March 19, 2025. Participants were provided reminder emails every other week (two reminders total) after the initial survey request to complete the survey. Participants who reported having a role with OPAT were eligible to answer the survey. Participants were asked to comment on OPAT as it occurs at the primary location where they work. The response rate was calculated based on the total number of EIN members who had ever answered a survey.

### Data analysis

Categorical data were presented using frequencies. For categorical variables, chi-squared or analyses of variance (ANOVA) were performed as applicable. Differences were considered statistically significant if *P* < .05. All quantitative analyses were performed using SAS v9.4 (SAS Institute Inc).

We also analyzed qualitative data. Participants were asked to respond to the item “Additional comments about OPAT and related safety issues” with free-text responses. Four authors (S.K., M.M., C.R, L.C.) systematically read the responses and derived codes. They reached agreement on classification of the responses. Percentages of respondents answering the item and illustrative quotes are presented.

## Results

Of 1639 active EIN clinicians who have ever responded to an EIN survey, 622 (38%) responded to this survey, of which 153 (25%) reported no role in OPAT and opted out of the survey. We analyzed results from the remaining 469 EIN members who completed the survey. The EIN member database includes member practice and employment data. Notably most respondents (180/469, 38%) worked at a university hospital, followed by community hospital (117/469, 25%), or non-university teaching hospital (116/469, 25%) (Table 1). Physicians comprised 95% (445/469) of respondents.

**Table 1.** Employment and OPAT structures as reported by infectious diseases clinicians in the emerging infections network

Characteristics	No. (%)
Employment	N = 469
University hospital	180 (38%)
Community hospital	117 (25%)
Non-university teaching hospital	116 (23%)
City/county hospital	28 (6%)
VA hospital or Department of Defense	25 (5%)
Outpatient only	2 (1%)
Role in OPAT^a^	N = 469
See patients receiving OPAT in clinic after hospital discharge	385 (82%)
Responsible for placing OPAT orders as inpatient consultant	327 (70%)
Recommend OPAT only as inpatient consult	189 (40%)
Manage OPAT program/clinic, or primary person responsible for managing OPAT	180 (38%)
Estimated number of OPAT patients followed per week	N = 462
<1–10	103 (22%)
11–50	201 (43%)
51–100	87 (19%)
101–200	58 (12%)
>200	13 (3%)

Note. ASP, Antimicrobial Stewardship program; ID, infectious diseases; OPAT, outpatient parenteral antimicrobial therapy, VA, veteran’s affairs.

a Respondents were able to select all responses that applied; numbers add to more than 100%.

**Figure 1.**
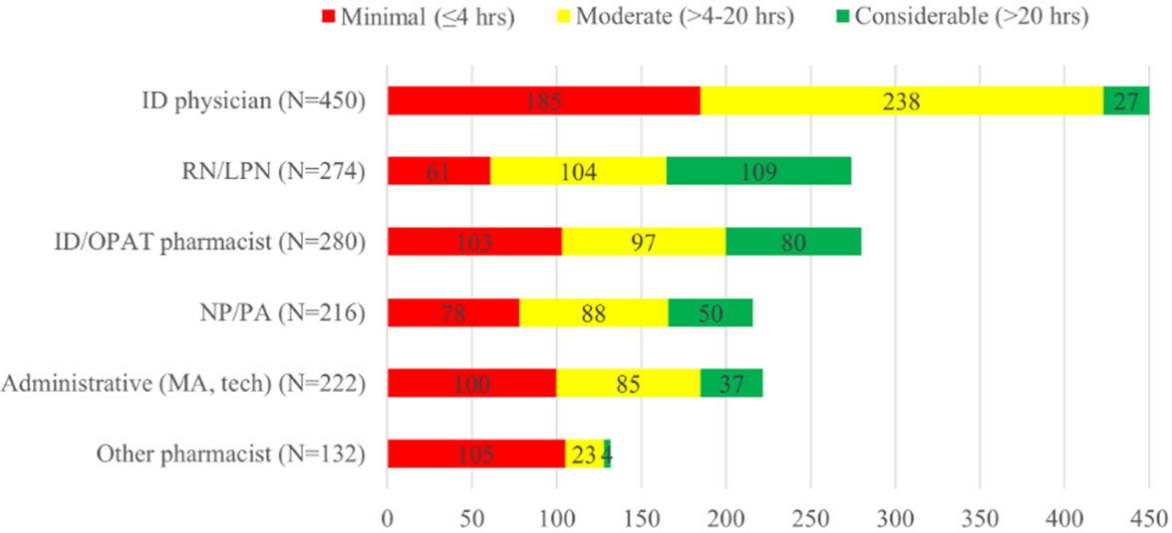
Respondents rated the amount of time spent in a usual week managing outpatient parenteral antimicrobial therapy patients by each of the health care workers listed. The numbers are the total number of participants who responded in each category. Each respondent could select more than one category of clinician involved in their program. ID, infectious diseases; LPN, licensed practical nurse; NP, nurse practitioner; OPAT, outpatient parenteral antimicrobial therapy; RN, registered nurse; PA, physician assistant.

While 189/469 respondents (40%) recommended OPAT only as an inpatient consultant, 385/469 respondents (82%) also saw OPAT patients in a clinic after hospital discharge (Table 1). Most OPAT programs were responsible for ≤50 patients a week (43%, 201/469, noted that their programs followed 11–50 OPAT patients per week; 22%, 103/469, followed <10 OPAT patients per week. ID physicians were most frequently involved in OPAT (noted by 450/469, 96%), followed by ID pharmacists (noted by 280/469, 60%), nurses (noted by 274/469, 59%), and APPs (noted by 216/469, 46%). Only 47% (222/469) of respondents had administrative support for OPAT (Figure 1). Of respondents who reported having multiple team members, 78% of respondents noted nurses, 64% reported APPs, 63% ID physicians, and 63% ID pharmacists were working at least 4hrs/week. Substantial proportions of respondents noted that dedicating over 20 hours per week on OPAT-related tasks occurred in various team members (nurses 40%, ID pharmacists 29%, APPs 23%, ID physicians 6%, administrative support 17%, other pharmacist 3%) (Figure 1).

**Figure 2.**
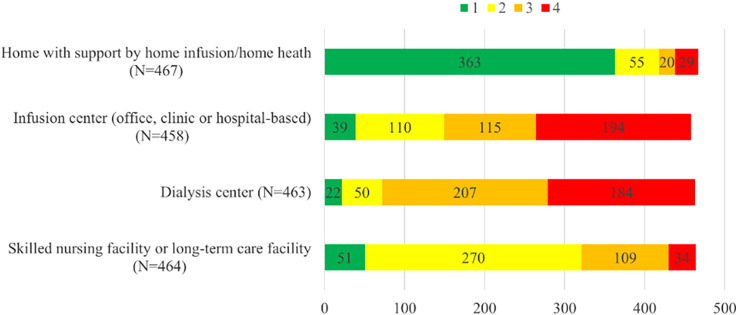
Respondents ranked outpatient parenteral antimicrobial therapy delivery sites from the most to least common where 1 is most Frequent and 4 is least frequent.

Most respondents (435/457, 95%) reported that their OPAT programs were managed by ID with majority reporting their OPAT programs required ID consultation prior to OPAT enrollment (280/469, 60%) (Table 2). Overwhelmingly, patients received antimicrobials at home, followed by receipt at a skilled nursing facility (SNF) or long-term care facility (Figure 2). Inclusion of COpAT (oral-only and/or long-acting injectable antimicrobial agents) in their OPAT program was reported by 59% of respondents. ID clinicians most often followed OPAT patients postdischarge, with the majority (380/469, 81%) of respondents indicating that >75% of OPAT patients were managed by ID. Outpatient ID physicians were routinely (349/464, 75%) responsible for receipt of and action on laboratory results. Multidisciplinary OPAT teams (173/464, 37%), OPAT pharmacists (152/464, 33%) and inpatient ID physicians (141/464, 30%) were also frequently involved. Having non-specialists (SNF clinician, discharging clinician/inpatient team, primary care clinician, other pharmacist) responsible for laboratory monitoring was reported by 32% (152/464). Laboratory values were most often available via fax/email (193/454, 43%) or by EHR (189/457, 41%); however, calling to request laboratory test results “often/always” was reported by 22% (101/450) of respondents.

**Table 2.** OPAT program details

Characteristics	No. (%)
ID consultation is required prior to OPAT enrollment	N = 469
Yes	280 (59.7%)
No	173 (36.9%)
Unsure	16 (3.4%)
OPAT program follows COpAT patients	N = 469
Yes	277 (59.1%)
No	183 (39.0%)
Not answered	9 (1.9%)
OPAT program oversight falls under	N = 457
Infectious diseases	435 (95%)
Pharmacy	108 (24%)
Hospital or ambulatory ASP	73 (16%)
Percentage of OPAT patients managed by infectious diseases^a^	N = 469
<25%	32 (7%)
26–50%	2 (0.4%)
51–75%	26 (6%)
>75%	380 (81%)
Not sure	29 (6%)
Labs followed by	N = 464
Outpatient ID physician	349 (75%)
Multidisciplinary OPAT program	173 (37%)
OPAT pharmacist	152 (33%)
Inpatient ID physician	141 (30%)
Skilled nursing facility clinician	79 (17%)
Discharging clinician/inpatient team	34 (7%)
Primary care clinician	30 (6%)
Stewardship physician	16 (3%)
Other pharmacist	9 (2%)

Note. OPAT, outpatient parenteral antimicrobial therapy; COpAT, complex antimicrobial therapy; ASP, Antimicrobial Stewardship program; ID, infectious diseases.

**Figure 3.**
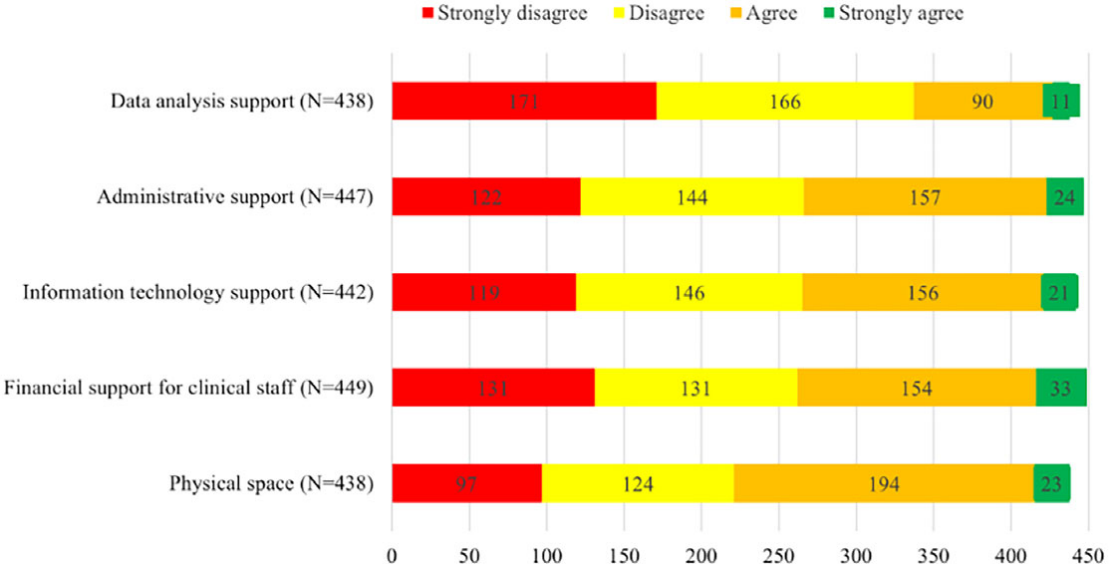
Respondents rated agreement with the following statement: “The OPAT program at my hospital receives adequate support in the following areas.”

**Figure 4.**
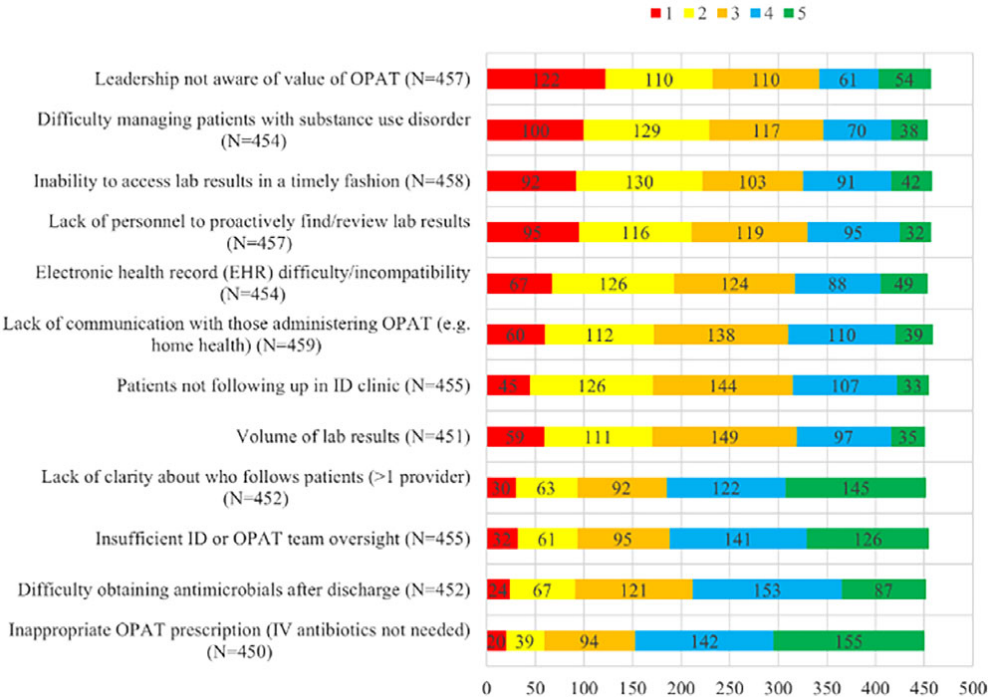
Barriers to safe OPAT care—factors present in providing safe OPAT services on a scale where 1 is “extremely challenging” and 5 is “not challenging at all.”

The majority did not feel that their OPAT programs had adequate support in terms of data analysis, administrative, information technology, and financial support for clinical staff (Figure 3). Only half the respondents stated they had adequate physical space. Additional barriers to providing safe OPAT care included lack of leadership awareness of the value of OPAT (232/457, 51%), difficulty managing patients with SUD (225/454, 50%), insufficient access to timely laboratory values (222/458, 48%), lack of personnel to retrieve missing laboratory values (211/457, 46%), and struggles with the EHR 193/454, (43%). Inappropriate OPAT enrollment (59/450, 13%), inability to obtain antimicrobials (91/452, 20%), and insufficient OPAT oversight (93/455, 20%) were less commonly identified as challenges (Figure 4).

Respondents were liberal with their free text comments with a total of 405 free text comments recorded throughout the survey, of which 103 pertained to additional comments about OPAT and related safety issues (Appendix 1). Several themes emerged from these 103 comments that focused on strategies for and barriers to safe OPAT care (Table 3). Development of a multidisciplinary team was frequently reported, and ranged from having a single nurse, to an ID pharmacist, and/or APP and technology/data analytics support for OPAT. Many comments acknowledged the shift toward oral antimicrobials, while also recognizing the gaps that have emerged in the shift to COpAT, such as lack of managing side effects and non-adherence. Better EHR support, including a desire to end the use of faxes, and trouble tracking down labs were recurring themes. Many comments also highlighted inadequate billing/reimbursement as well as need for more institutional support. Interestingly, many respondents specifically detailed the challenges in following patients in SNFs, with difficulty communicating with SNFs, tracking down labs, and patients not attending outpatient appointments. Some programs reported that they did not follow patients discharged to SNFs. Some respondents felt that their OPAT programs functioned well and noted facilitators including institutional support and a multidisciplinary team.

**Table 3.** Select free-response comments about provision of OPAT

Comments theme(s)	Illustrative quotes
Better EHR support	Labs faxed are problematic- they don’t show trends easily. Also signing OPAT orders for infusion companies are frustrating—have to fax them back. Obtaining labs from HD centers is extremely challenging. At present our preferred infusion vendor does not have read only access to EPIC which would enable communication about labs.
COpAT	In the move to COpAT, there is very little recognition on the part of physicians (both ID and non-ID) on the challenges in managing patients on oral antibiotics for long periods of time. This includes side effects, non-adherence due to co-pays, etc. These are issues that are often picked up an OPAT nurse/pharmacist, but there are no safeguards built in for patients on COpAT. OPAT is far less relevant to our institution than in the past given heavy favoritism towards evidence-based oral transition therapy
Inadequate billing/reimbursement	Benchmarking optimal OPAT staffing ratios to patient volume; novel reimbursement models; telehealth coverage. There needs to be advocacy/legislation so insurers reimburse non-face-to-face work done on OPAT which is massive. The current non-face-to-face billing mechanism is insanely difficult, poorly reimbursed and not something we pursue so we get inadequate hospital support and struggle to keep up with the volume. We give away so much uncompensated time.
Need for more institutional support	Hospital administration does NOT recognize the work that ID does with OPAT, does not take this into account when they calculate “RVU targets”, a lot of non-reimbursed work, other services especially surgical services feel ID should just arrange all the OPAT as outpatient with the limited resources we have. It would be very helpful to have standard/published staffing ratios to help justify our requests for more staffing to hospital leadership
Trouble tracking down labs	In a private practice where we manage all of our OPAT. It is not a formal program through the hospital. Other providers would have ability to order outpatient IV antibiotics but always involve ID to recommend and manage. Obtaining labs from outside home cares or facilities can be challenging and not always done as ordered. Our OPAT care manager (RN) spends so much time chasing down labs. We cover a very large geographic area - so many different facilities, ways to obtain labs, everyone has their own order forms they want. Our nurse does a phenomenal job obtaining labs/orders and making sure we don’t lose patients but it is a full time job with a lot of babysitting. When she has time off we struggle.
Challenges in SNF	Our OPAT service decided to stop managing OPAT in SNF patients due to potential liability and challenges. However, I think this is deeply unethical as these are the patients with the highest risk of harm, and the most oversight needed. For us, nursing homes are the largest area of concern. Labs either never done, or done but never reported. Patients who either show up very late, or not at all (from the ECF) [extended care facility].
Good system/things work/functioning	Our program has improved with increasing support from the institution, it was a long process but successful. We have a multidisciplinary OPAT team consisting of an ID pharmacist, RN, and administrative assistant that significantly reduces the burden on physicians and has dramatically reduced readmissions since its inception.

Note. EHR, electronic health record; HD, hemodialysis, COpAT, complex antimicrobial therapy; RVU, relative value unit; RN, registered nurse; SNF, skilled nursing facility; ECF, extended care facility.

## Discussion

In this survey, one of the first to survey a broad national sample of not only ID physicians but also ID pharmacists and APPs about OPAT, we demonstrate the importance of multidisciplinary teams in the management of OPAT patients and the new incorporation of oral and long-acting injectable antimicrobials into OPAT programs. However, as in previous surveys, many barriers to the safe delivery of OPAT remain.

Notably, barriers to safe care that most respondents reported as “extremely challenging” included a lack of leadership awareness of the value of OPAT, management of patients with SUD, and inability to access laboratory test results. In addition, multiple challenges in communicating with postacute care locations were reported, such as lack of personnel to proactively identify and review laboratory test results, EHR incompatibility, and lack of communication with those administering OPAT. Challenges such as follow-up of patients in postacute care, communication with those administering OPAT (eg, home health organizations, hemodialysis facilities, and SNFs), patients not following up in ID clinic, and lack of clarity about who follows patients were themes in the survey and comments, and were also major barriers noted in prior surveys regarding OPAT practices.^17,18^

Our findings suggest that OPAT team composition continues to expand. Fewer respondents indicated that ID physicians spent >20 hours per week on OPAT-related work than in prior surveys, while more respondents indicated that other groups spent >20 hours per week on OPAT-related work than in prior surveys. Comparing 2018 survey results to 2025 numerically among respondents reporting >20 hours per week on OPAT-related work, ID physician time decreased from 10.5% to 6.0%, while ID pharmacists’ increased from 11.7% to 28.6%, nurses from 26.3% to 39.8%, and APPs from 9.1% to 23.2%.^17^ This is reassuring, as literature demonstrates that use of multidisciplinary OPAT teams can lead to improved outcomes.^20,21^

While multidisciplinary growth in OPAT programs is encouraging, studies show that OPAT is a resource intensive endeavor, and much of the work is uncompensated.^22,23^ Additionally, 59% of respondents reported following patients on COpAT. While COpAT is cost effective and safe,^24^ and can further decrease healthcare costs,^25^ there is increasing recognition of the need for close follow-up,^26^ with wide variation in the recognition by both physicians and programs of the work involved.^27^ Several comments alluded to the lack of a definition of COpAT, as well as lack of standardized practices regarding follow-up of patients on COpAT.

Most patients received OPAT at home, followed by SNFs. However, an additional theme that emerged from the comments was the lack of oversight of patients receiving OPAT in SNFs or at hemodialysis. While our survey did not specifically ask about follow up of patients in SNFs, prior literature has shown that patients receiving OPAT in SNFs without specific OPAT team oversight tend to have poorer outcomes,^28^ whereas programs that do follow patients in SNFs note that patients discharged to home versus SNFs have similar outcomes.^23^ Several barriers to following these patients included poor communication, difficulty tracking laboratory test results, and challenges with transportation to follow-up outpatient specialty appointments. In addition, post-COVID era declines in SNF staffing may contribute to some of these outcomes.^29^

Our survey has limitations. The response rate was 38%, though overall numbers of respondents was comparable to prior EIN surveys about OPAT, and similar to response rates from other clinician surveys.^30,31^ The majority of survey respondents were physicians, with some pharmacists and APPs, however nurses were not included in the survey, even though they often dedicate the most time per week to OPAT activities. As OPAT programs continue to grow and evolve, future surveys should include all stakeholders who contribute to the successful implementation of OPAT. Additionally, not all respondents have a dedicated OPAT program. Indeed, many variations in practice set-up were reported, ranging from small practices that follow <10 OPAT patients per week, to large multidisciplinary institutional programs that manage >200 patients per week. This highlights the need to develop a framework that incorporates a range of practice strategies that can be adapted by different practitioners, recognizing that ultimately, the best OPAT program is one that can be adapted for a given practice situation.

## Conclusion

While lack of institutional support remains a barrier, our findings suggest the need to develop guidance for optimal staffing ratios, as has been proposed by others^12,15^ and development of a definition for COpAT.^32^ Recognition by leadership of the value added by COpAT care, changes to reimbursement models and strategies to overcome communication barriers and modernize systems to track lab results, are paramount to improved patient outcomes. Toward that end, our findings suggest the need to address operational and legal/compliance barriers, as well as changes in healthcare policy, to encourage the adoption of newer technologies, such as EHR integration to manage laboratory test results, use of artificial intelligence tools to eliminate manual review of data and identify patients at risk for adverse events, and expansion of telehealth to improve patient follow-up, including for those in SNFs.

## Supporting information

10.1017/ash.2026.10347.sm001 Castellino et al. supplementary material Castellino et al. supplementary material

## Supplementary material

The supplementary material for this article can be found at https://doi.org/10.1017/ash.2026.10347.
