# Supplementary material for: Management of outpatient parenteral antibiotic therapy: a United States-based multi-center survey
Source: Antimicrob Steward Healthc Epidemiol. 2026 Apr 17;6(1):e109. doi: 10.1017/ash.2026.10347 (PMC13104569; doi:10.1017/ash.2026.10347)
Supplement: Castellino et al. supplementary material [file S2732494X26103477sup001.pdf]

INFECTIOUS DISEASES SOCIETY OF AMERICA EMERGING INFECTIONS NETWORK QUERY:

Management of Outpatient Parenteral Antimicrobial Therapy (OPAT)

Over the last twenty years, the numbers of patients on outpatient parenteral antimicrobial therapy (OPAT) and complex outpatient antimicrobial therapy (COpAT) have increased dramatically. Understanding how to safely monitor these patients is essential.

In a 2018 EIN survey, only 36% of respondents noted they had a formal OPAT program to monitor patients, despite OPAT programs being recommended (Handbook of Outpatient Parenteral Antimicrobial Therapy for Infectious Diseases https://www.idsociety.org/opat-ehandbook/). We would like to understand how the practice of OPAT monitoring and care has changed. We would also like to learn about barriers ID clinicians continue to face as they help ensure their patients receiving OPAT are safe.

The goal of this survey is to learn how OPAT programs across the country are structured and how clinicians handle potential safety issues for patients on OPAT.

Please fax to 319-384-8860 OR 319-384-7208

EMERGING INFECTIONS NETWORK QUERY
Management of Outpatient Parenteral Antibiotic Therapy (OPAT)

Name: ________________________________

1. What is your role with OPAT? *[Please select all that apply]*
- __ No role (do not care for patients on OPAT) – STOP HERE and submit survey
- __ Recommend OPAT only as inpatient consultant
- __ Responsible for placing OPAT orders as inpatient consultant
- __ See patients receiving OPAT in clinic after hospital discharge
- __ Manage OPAT program or clinic, or primary person responsible for managing OPAT

Comments / Other role:

2. Is an ID consultation required for outpatient IV antibiotic (OPAT) prescription?

__ Yes __ No __ Not sure

3. What proportion of OPAT patients are followed by ID rather than non-ID practitioners?

__ <25% __ 26-50% __ 51-75% __ >75% __ Not sure

4. Approximately how many OPAT patients do you or your OPAT program follow per week?

__ <1-10 __ 11-50 __ 51-100 __ 101-200 __ >200

5. Please rank order where your patients receive OPAT: *[Rank from 1 (most frequent) to 4 (least frequent)]*
- __ Home with support from home infusion / home health
- __ Infusion center (office, clinic, or hospital-based)
- __ Dialysis center
- __ Skilled nursing facility or long-term care facility

Comments / Other location:

6. When patients are discharged on IV antibiotics, who is usually responsible for monitoring and acting upon lab results? *[Select all that apply]*
- __ Outpatient ID physician
- __ Inpatient ID physician
- __ Discharging provider/inpatient team
- __ Primary care provider
- __ Multidisciplinary OPAT program
- __ Skilled nursing facility provider
- __ Stewardship physician
- __ OPAT pharmacist
- __ Other pharmacist

Comments / Other:

7. Please categorize type and amount of clinician effort per week in the OPAT Program: *[Leave blank if job category not applicable]*

	Minimal (≤4 hrs)	Moderate (>4-20 hrs)	Considerable (>20 hrs)
ID physician	1	2	3
NP/PA	1	2	3
ID/OPAT pharmacist	1	2	3
Other pharmacist	1	2	3
RN/LPN	1	2	3
Administrative (MA, tech)	1	2	3

Please fax to 319-384-8860 OR 319-384-7208

8. Please rate your agreement with the following statement:
The OPAT program at my hospital receives adequate support in the following areas:

	Strongly disagree	Disagree	Agree	Strongly agree
Financial support for clinical staff				
Administrative support				
Information technology support				
Data analysis support				
Physical space				

9. Are patients on oral-only high-risk antimicrobials or long-acting injectable agents (COpAT) followed by your OPAT program?
__ No __ Yes, please provide examples:

10. Oversight of your OPAT program falls under: *[Please select all that apply]*
__ Hospital or ambulatory antimicrobial stewardship program (ASP)
__ Infectious diseases
__ Pharmacy
Comments or other oversight:

11. Please rate the challenge these factors present in providing safe OPAT services on a scale where 1 is "extremely challenging" and 5 is "not challenging at all":

Leadership not aware of value of OPAT	1 2 3 4 5
Inability to access lab results in a timely fashion	1 2 3 4 5
Lack of communication with those administering OPAT (e.g. home health)	1 2 3 4 5
Volume of lab results	1 2 3 4 5
Lack of personnel to proactively find/review lab results	1 2 3 4 5
Electronic health record (EHR) difficulty / incompatibility	1 2 3 4 5
Patients not following up in ID clinic	1 2 3 4 5
Lack of clarity about who follows patients (>1 provider)	1 2 3 4 5
Difficulty obtaining antimicrobials after discharge	1 2 3 4 5
Inappropriate OPAT prescription (IV antibiotics not needed)	1 2 3 4 5
Insufficient ID or OPAT team oversight	1 2 3 4 5
Difficulty managing patients with substance use disorder	1 2 3 4 5

Comments or other barriers in your practice:

12. How frequently do you receive laboratory results on OPAT patients via:

	**Never/Rarely**	**Sometimes**	**Often/Always**
EHR automatic availability	1	2	3
Fax / email to your office	1	2	3
Receipt after call to request results	1	2	3
Do not receive lab results	1	2	3

13. Additional comments about OPAT and related safety issues, or need for further research:

Thank you for completing this survey!

Please fax to 319-384-8860 OR 319-384-7208
